# The Transient Receptor Potential Vanilloid Type 2 (TRPV2) Channel–A New Druggable Ca^2+^ Pathway in Red Cells, Implications for Red Cell Ion Homeostasis

**DOI:** 10.3389/fphys.2021.677573

**Published:** 2021-06-10

**Authors:** Stéphane Egée, Lars Kaestner

**Affiliations:** ^1^Sorbonne Université, CNRS, UMR 8227, Integrative Biology of Marine Models, Station Biologique de Roscoff, Roscoff Cedex, France; ^2^Laboratoire d'Excellence GR-Ex, Paris, France; ^3^Theoretical Medicine and Biosciences, Saarland University, Saarbrucken, Germany; ^4^Experimental Physics, Saarland University, Saarbrucken, Germany

**Keywords:** red cells, ion channels, Ca^2+^ signaling, volume regulation, storage lesions

## Introduction

Ca^2+^-permeable channels in red blood cells (RBCs) is a timely topic (Filser et al., 2020; Wang et al., 2021). They are of particular importance because the intracellular Ca^2+^ seems to be a major mediator in numerous RBC-related diseases including sickle cell disease (Mahkro et al., 2020) and numerous unrelated rare hereditary anemias (Hertz et al., 2017). Saying this, the focus (almost a hype) of the past years was on the mechanosensitive channel PIEZO1 since its discovery in 2010 (Coste et al., 2010) and especially the association of mutations in this channel with hereditary xerocytosis (Zarychanski et al., 2012; Albuisson et al., 2013; Andolfo et al., 2013; Bae et al., 2013; Rotordam et al., 2018). However, the entire picture of Ca^2+^ regulation in general and Ca^2+^-permeable channels in particular is a lot more versatile (Kaestner et al., 2020).

## The TRPV2 Channel–Questions In Relation To The Properties Reported In RBCs

Very recently the transient receptor potential vanilloid type 2 (TRPV2) channel was reported to be present in RBCs (Belkacemi et al., 2021). TRPV2 is a non-selective cation channel conducting also Ca^2+^ and can be activated by Δ9-tetrahydrocannabinol (Δ9-THC) or cannabidiol (CBD). This is a milestone in RBC electrophysiology but at the same time raises a number of questions in the context of the channels physiological function and RBC hydration status, a discussion we like to stimulate with this opinion paper.

The discovery of the TRPV2 in RBCs is a remarkable finding because the abundance of ion channel copies is very low in the RBC membrane and functional channel recordings are complicated to align with molecular identities (Kaestner, 2015). Even in the study, where the TRPV2 was found, the detection of the Gárdos channel (KCNN4, K_Ca_3.1, hSK4) was below the quality threshold in the proteomic study (Belkacemi et al., 2021).

We like to discuss two particular outcomes in more detail that may be relevant for the understanding of RBC physiology. A key point of the report by Belkacemi et al. (2021) was an increase of the osmotic fragility in TRPV2 KO mouse RBCs. The conclusion drawn is that TRPV2 mediated Ca^2+^ entry activates the Gárdos channel followed by K^+^ loss and subsequent loss of water, similar to what has already been suggested for PIEZO1. If this is the case one would expect cell shrinkage/dehydration upon TRPV2 activation with the channels agonists Δ9-THC and CBD, but the authors found in agreement with previous investigations (Chari-Bitron and Shahar, 1979) a cell swelling/overhydration. The explanation of this effect remains completely elusive, although we propose an initial concept of a mechanism (see below: section Putative consequences of TRPV2 activation in RBC after cannabis consumption).

In their paper (Belkacemi et al., 2021) showed that osmotic fragility is decreased upon RBC stimulation with Δ9-THC with Ca^2+^ being present in the external solution, that could partly be reversed by the additional application of the Gárdos channel inhibitor TRAM34. However, if RBCs are exposed to hypoosmotic conditions as performed in the paper, the cell swelling is expected to activate PIEZO1 or other mechanosensitive channels that mediate Ca^2+^ influx (Danielczok et al., 2017) i.e., one would expect a Gárdos channel-dependent modulation of the osmotic fragility since internal Ca^2+^ should increase, mediated by mechanosensitive cation channel activity. Surprisingly and for unknown reasons TRAM34 alone had no effect on the osmotic tolerance.

## A Putative Contribution of TRPV2 in RBC Storage Lesions

If new channels are identified to be present in the RBCs membrane at the protein level, the question arises, if previous functional reports of channel activity can be aligned with the molecular discovery which is a complicated task (Kaestner, 2015).

**Figure 1:**
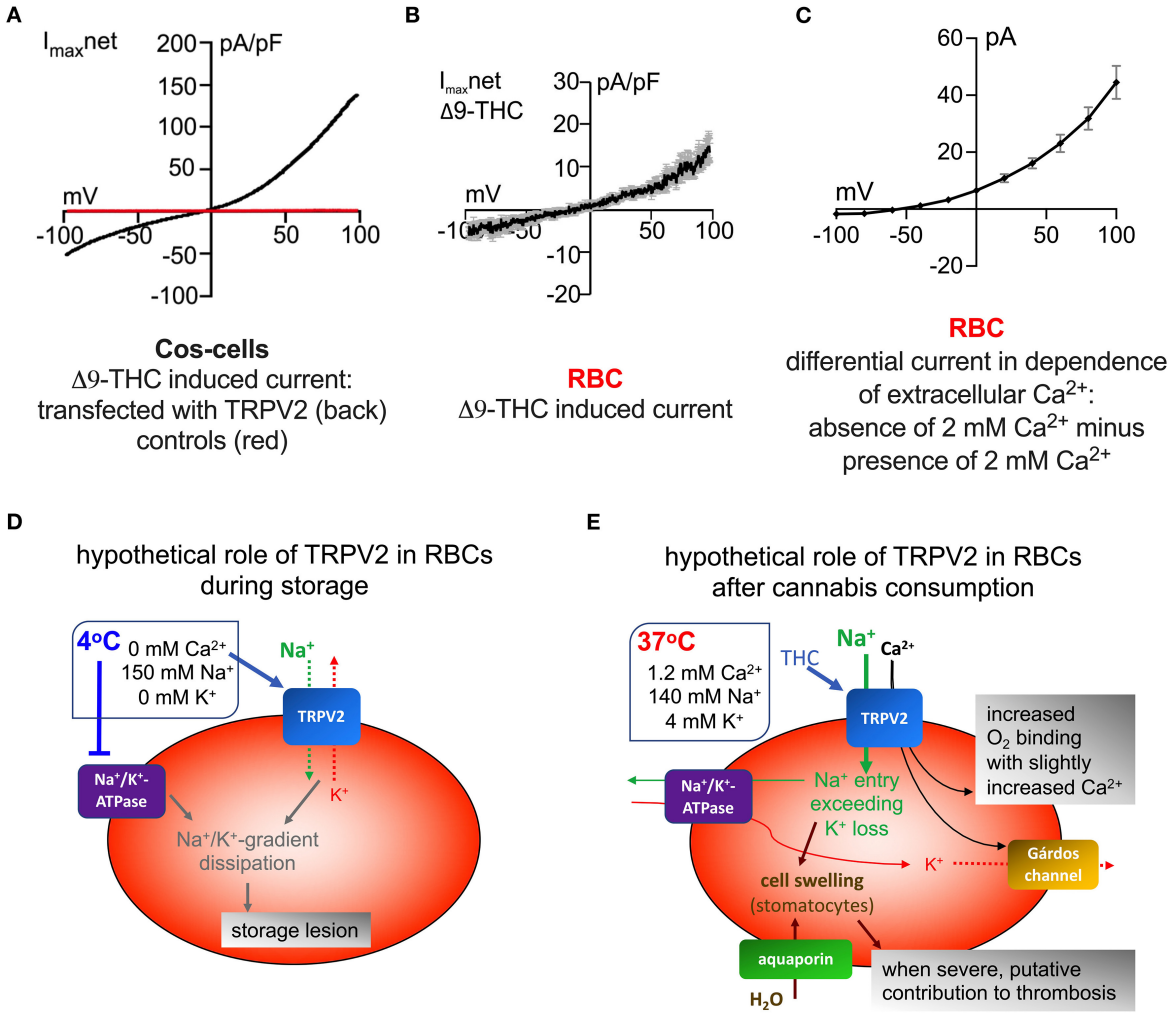
Comparison of I-V behavior of ion channels. **(A)** Whole cell currents measured in Cos-cells overexpressing TRPV2 (black trace) and control cells (red trace), both stimulated with 30 μM Δ9-THC. The internal solution contained (in mM): 120 Cs-glutamate, 8 NaCl, 1 MgCl_2_, 10 HEPES, 10 Cs-BAPTA, 3.1 CaCl_2_ (corresponding to100 nM free Ca^2+^), pH adjusted to 7.2 with CsOH, and the external solution contained (in mM): 140 NaCl, 2 MgCl_2_, 1 CaCl_2_, 10 HEPES, 10 glucose, pH adjusted to 7.2 with NaOH. For TRPV2 stimulation Δ9-THC was added to the external solution and applied directly to the patch clamped cell via an application pipette. Voltage ramps of 400 ms duration spanning a voltage range from −100 to 100 mV were applied at 0.5 Hz from a holding potential of 0 mV over a period of 300–400 s. **(B)** Whole cell currents measured in RBCs stimulated with 30 μM Δ9-THC. Recording conditions were identical to the ones described in **(A)**. **(C)** Differential whole cell current measured in RBCs when 2 mM Ca^2+^ is removed from the extracellular solution. The current recorded in 2 mM CaCl_2_-external solution was subtracted from the current recorded in 0 mM CaCl_2_ -external solution. The internal solution contained (in mM): 50 CsCl, 20 NaCl, 60 CsF, 5 MgATP, 10 HEPES, 20 EGTA, pH adjusted to 7.2 with CsOH, and the external solution contained (in mM): 0 or 2 mM Ca^2+^, 125 TEACl, 10 HEPES, 5 MgCl_2_, 45 glucose, pH adjusted to 7.3 with TEA-OH. Please note that tetraethylammonium (TEA) is non-permeant to most cation channels and therefore cationic outward currents (corresponding to negative membrane potentials) are not expected. For the same reason, the reversal potential (membrane potential when no currents occur–intersection of curve with x-axis) is expected to be shifted toward more negative values. Recordings were conducted using voltage steps from −100 to 100 mV for 500 ms in 20 mV increments at 5 s intervals, the holding potential being set at −30 mV. In **(A,B)** the current is normalized to the cell capacitance (pA/pF), while in **(C)** the authors felt that in the planar chips, the capacitance does not correctly reflect the cell surface and therefore just provided the current per cell recording (pA). Additionally, the recording protocols were completely different, for **(A,B)**, voltage ramps were applied and for **(C)** discreate voltage steps were recorded to compile the I-V relationship. **(A,B)** are reprints with permission from (Belkacemi et al., 2021) and **(C)** is reproduced from (Petkova-Kirova et al., 2018). **(D)** Suggested channel activity in RBCs during storage. The rectangle next to the RBC describes the main components of typical storage conditions. **(E)** Putative membrane transport interactions in RBCs after cannabis consumption. The rectangle next to the RBC describes the main components relevant for cation transport in the blood plasma.

TRPV2, like many members of the TRP family, shows a Ca^2+^-dependent desensitization, inhibition, or inactivation by extracellular Ca^2+^. More importantly, this function is maintained albeit TRPV2 does not contain the binding sites for calmodulin (CaM), adenosine triphosphate (ATP), or phosphatidylinositol-4,5-bisphosphate (PIP_2_) (Mercado et al., 2010). Such a behavior echoes a recent report of an unrecognized non-selective cation channel in human RBCs activated upon extracellular Ca^2+^ depletion and thought to potentially be a non-negligible part of the leaky pathways that contribute to the cation gradients dissipation upon storage with Ca^2+^ depleted solutions (Petkova-Kirova et al., 2018). To illustrate the similarity in functional behavior, we compiled Figures 1A–C to compare the I-V behavior of TRPV2 as described by Belkacemi et al. (2021) and the non-selective cation channel reported by Petkova-Kirova et al. (2018). When comparing the I-V relationship one should keep in mind the different recording conditions in two different laboratories. Furthermore, the ionic composition of both the internal and external solution differs as detailed in the figure legend. All these differences may explain particular deviations between the curves. Overall they look very similar (outward rectified), what is compatible with an agreement of TRPV2 (Belkacemi et al., 2021) and the channel activated by Ca^2+^ removal (Petkova-Kirova et al., 2018). However, this is by far not a proof. Therefore, future investigations need to confirm that TRPV2 is involved in RBC storage lesions. Nevertheless, for illustrative purposes, in Figure 1D we provide a scheme of the hypothesized contribution of TRPV2 to RBC cation gradient dissipation in storage lesions. We maintain the vision to have with TRPV2 a molecular player that could be pharmacologically addressed and potentially improve RBC storage conditions.

## TRPV2 IN RBC Physiology and Pathophysiology

We like to raise another aspect that is likely to be important to judge the relevance of TRPV2 in RBC. TRPV2 was reported to be a mechanosensitive channel (Muraki et al., 2003), and although we have no data on this mechanic activation in RBCs it is an appealing concept that TRPV2 could act similar as PIEZO1 and under certain conditions even compensate impaired PIEZO1 activity or may have in normal conditions a complimentary function. To this end we provide Table 1, comparing characteristic properties of TRPV2 and PIEZO1. Indeed, the PIEZO1 channel has a very particular activation signature. The range of pressure (or membrane tension) necessary for its activation is absolutely compatible with those encountered in the circulation or even in the passage of the splenic filtration. After opening the channel closes extremely quickly, probably rendering the increase in Ca^2+^ most of the time inoperative for a significant activation of the Gárdos channel leading to an alteration in cell volume (Please note that the PIEZO1 opening kinetics upon mechanical stimulation is different to activation by its agonist Yoda1). Indeed, the propensity of Ca^2+^ to be an efficient effector of RBC homeostasis is not only linked to its capacity to enter cells rapidly via conductive pathways, but also to the capacity of the plasma membrane Ca^2+^ ATPase (PMCA) to counterbalance this massive influx. Therefore, the PMCA limits the irreversibility of the subsequent activation of the Gárdos channel (Lew and Tiffert, 2017). This last point is of high interest regarding RBCs homeostasis, since Ca^2+^ flux plays most of the time the pivotal role, both in RBC physiology and pathophysiology. Such the Ca^2+^ homeostasis is also linked to RBC metabolism.

**Table 1:** Comparative summary features of TRPV2 channels *vs*. PIEZO1 channels.

**Property**	**TRPV2**	**PIEZO1**	**References**
Structure	Homotetramer	Homotrimer	Ge et al., 2015; Muller et al., 2019
Selectivity	Ca^2+^ > Mg^2+^ > Na^+^ ~ Cs^+^ ~ K^+^; P_Ca_/P_Na_ = 2.94; P_Mg_/P_Na_ = 2.40	P_K_ > P_Cs_ ≈ P_Na_ > P_Li_ (1.0:0.88:0.82:0.71) significant permeability for Ca^2+^, Mg^2+^, and Ba^2+^	Perálvarez-Marín et al., 2013; Gnanasambandam et al., 2015
Temperature	>52°C	No incidence	Perálvarez-Marín et al., 2013
Mechanoactivation	Stretch, shear stress, hypotonicity (P_50_ ≈ 50 mmHg)	Stretch, shear stress, hypotonicity (P_50_ ≈ 30 mmHg)	Coste et al., 2012; Moore and Liedke, 2017
I/V curve	Outwardly rectifying	Linear	Gnanasambandam et al., 2015; Moore and Liedke, 2017
Unitary conductance	20–40 pS	35–55 pS	Gnanasambandam et al., 2015; Zhang et al., 2016
Kinetic properties	No inactivation	Fast deactivation (ms range)	Coste et al., 2012
Agonists	*Cannabis sativa* derivatives (EC_50_ μM range)	YODA1, JEDI1/2 (EC_50_ from nM to μM range)	Neeper et al., 2007; Lacroix et al., 2018
Antagonists	SKF96365 and amiloride ruthenium red, trivalent cations (IC_50_ μM range)	GsMTx4, trivalent cation, ruthenium red (IC_50_ μM range)	Bae et al., 2011; Moore and Liedke, 2017

*Please note that most of the channel properties summarized here do not originate from RBC measurements*.

Considering that with PIEZO1 and TRPV2, two mechanically sensitive channels carrying Ca^2+^ has a definite advantage, allowing for finer modulation to ensure Ca^2+^ signaling which, if left unchecked, can rapidly alter the filterability of RBCs and lead to a reduction in their lifespan within the circulation. However, it is completely elusive if putative genetic variants of TRPV2 are molecular contributors to hereditary xerocytosis or any other hemolytic anemias.

Moreover, one of the aspects often overlooked when considering the effects of Ca^2+^ influx via conductive pathways into RBCs is the direct impact on the membrane potential. A Ca^2+^ entry, even minimal, will immediately cause a substantial depolarization of the RBCs from a resting membrane potential of ~-12 mV even before secondary effects like the activation of the Gárdos channel take place. This is not negligible and could explain some unexplained experimental observations (Kaestner et al., 2018; Jansen et al., 2021).

## Putative Consequences of TRPV2 Activation in RBC After Cannabis Consumption

Since TRPV2 is activated by the cannabinoids Δ9-THC and CBD one can expect a TRPV2 activation in RBCs after cannabis consumption. In Figure 1E we sketched the molecular interactions of membrane transport proteins as we would expect from the known RBC membrane transport functions (Bernhardt and Ellory, 2003) and the observation that exposure of RBCs to Δ9-THC results in cell swelling (Chari-Bitron and Shahar, 1979; Belkacemi et al., 2021). We hypothesize two distinct effects. If there is only a slight activation of TRPV2, a minor increase in the RBC Ca^2+^ concentration could be stimulating and advantageous toward an increased oxygen binding affinity (Makhro et al., 2013). In contrast, a strong activation of TRPV2 leading to the afore mentioned cell swelling is likely to impair the capillary flow, where the RBC diameter exceeds the vessel diameter (Kihm et al., 2021). The cannabinoid induced increase in RBC volume could very well-contribute to the thrombotic events, frequently reported to occur after cannabis consumption especially in combination with vasoconstriction that is also related to cannabis consumption (Disdier et al., 2001; Mittleman et al., 2001; Peyrot et al., 2007; Wolff et al., 2011).

The molecular regulation as depicted in Figure 1E is a bit more complicated as the scheme in Figure 1D and is not limited to the Ca^2+^ permeability of TRPV2. Indeed, an increase in the intracellular Na^+^ concentration seems the only plausible explanation for the RBC swelling and as a consequence the Na^+^ influx must exceed the K^+^ efflux. This means the Na^+^ influx via TRPV2 must exceed the K^+^ efflux by the Gárdos channel, especially since the Na^+^/K^+^-ATPase has a Na^+^:K^+^ stoichiometry of 3:2. However, a variable abundance ratio of TRPV2 and Gárdos channel in individual RBCs could well explain the cellular variability in the RBC hydration state after Δ9-THC stimulation.

## Conclusion

The biochemical data of the recently reported abundance of the TRPV2 in RBCs are sound and convincing (Belkacemi et al., 2021). However, further research on the functional properties of TRPV2 in RBCs and the involvement of TRPV2 in: (i) RBC physiology inclusive effects caused by cannabis consumption, (ii) the genesis of RBC related disease, (iii) in the treatment of malaria as proposed by the authors in the original report and (iv) its contribution to the cation gradients dissipation upon storage as outlined above, are now required.

## Author Contributions

SE and LK wrote the manuscript and agree to be accountable for the content of the work.

## Conflict of Interest

The authors declare that the research was conducted in the absence of any commercial or financial relationships that could be construed as a potential conflict of interest.
